# Psychological illness is commonly associated with functional gastrointestinal disorders and is important to consider during patient consultation: a population-based study

**DOI:** 10.1186/1741-7015-3-8

**Published:** 2005-05-13

**Authors:** Ture Ålander, Kurt Svärdsudd, Sven-Erik Johansson, Lars Agréus

**Affiliations:** 1 Family Medicine, Stockholm, Karolinska Institute, Sweden; 2 Ålander Family Practice, Uppsala, Sweden; 3 Department of Public Health and Caring Sciences, Unit of Family Medicine, University Hospital, Uppsala, Sweden

## Abstract

**Background:**

Some individuals with functional gastrointestinal disorders (FGID) suffer long-lasting symptoms without ever consulting their doctors. Our aim was to study co-morbidity and lifestyle differences among consulters and non-consulters with persistent FGID and controls in a defined adult population.

**Methods:**

A random sample of the general adult Swedish population was obtained by a postal questionnaire. The Abdominal Symptom Questionnaire (ASQ) was used to measure GI symptomatology and grade of GI symptom severity and the Complaint Score Questionnaire (CSQ) was used to measure general symptoms. Subjects were then grouped for study by their symptomatic profiles. Subjects with long-standing FGID (n = 141) and subjects strictly free from gastrointestinal (GI) symptoms (n = 97) were invited to attend their local health centers for further assessment.

**Results:**

Subjects with FGID have a higher risk of psychological illness [OR 8.4, CI_95_(4.0–17.5)] than somatic illness [OR 2.8, CI_95_(1.3–5.7)] or ache and fatigue symptoms [OR 4.3, CI_95_(2.1–8.7)]. Subjects with psychological illness have a higher risk of severe GI symptoms than controls; moreover they have a greater chance of being consulters. Patients with FGID have more severe GI symptoms than non-patients.

**Conclusion:**

There is a strong relation between extra-intestinal, mental and somatic complaints and FGID in both patients and non-patients. Psychological illness increases the chance of concomitantly having more severe GI symptoms, which also enhance consultation behaviour.

## Background

Gastrointestinal (GI) symptoms are common and are reported within three months by almost every other adult in Western countries (UK, Sweden, USA, Australia) [1-5]. (The abbreviations used in this manuscript are explained in the Additional File.) The most common symptoms are gastroesophageal reflux symptoms/disease (GERS) and dyspepsia originating from the upper GI tract, and Irritable Bowel Syndrome (IBS) from the lower GI tract. Prevalence rates are reported to be 25% for both GERS and dyspepsia and about 12% for IBS [6]. Although intermittent, these disorders may linger in many sufferers [2,7]. The total cost of dyspepsia to society, with and without peptic ulcer disease, GERS and IBS, is considerable [8,9]. Thus, these disorders constitute a major public health problem.

Dyspepsia without peptic ulcer disease (PUD) (or any other organic GI disease) is called functional dyspepsia [10]. In a Swedish population-based upper endoscope study, the prevalence of dyspepsia was 38% and the prevalence of PUD 4%, with about a quarter of the PUD subjects having no GI symptoms [48]. Thus, the vast majority of those reporting dyspepsia in the population can be expected to have functional dyspepsia. IBS is a functional gastrointestinal disorder [11]. Although functional dyspepsia and IBS are considered separate disorders [10,12], many subjects report concomitant symptoms thought to originate from both the upper and the lower the GI tract [1,13]. Also, sufferers may experience a change in predominant symptom profile over time between dyspepsia and IBS, but much less towards GERS [2]. This means that dyspepsia and IBS sufferers probably have common aspects of both pathophysiology and health care seeking behavior. Furthermore GERS, in a majority of cases, has a proven (non-functional) acid-related etiology [11]. Accordingly, it seems reasonable to exclude subjects with only GERS symptoms from the functional definition and to investigate the burden on society of functional dyspepsia and IBS taken together. We thus label these conditions as functional gastrointestinal disorders or FGID.

A minority of subjects with FGID (5–20%) are reported to visit a doctor quarterly for their complaints [14,15]. On the other hand, up to half of those with FGID never see a doctor despite prolonged symptoms [14,16]. For IBS, the proportion is even higher, up to 80 % [14]. Thus, GI patient-based data will not cover all aspects of morbidity. Moreover, it has been reported previously that most patients' sick leave is due to disorders other than their abdominal complaints. Only 23% of those subjects with abdominal pain who were on sick leave stated that abdominal pain was the cause of their absenteeism [17].

There are prior cross-sectional population-based point prevalence studies on GI co-morbidity [18-20]. However, there is a lack of knowledge of the co-morbidity of subjects with proven chronic/long lasting/persistent FGID and its impact on health care seeking behaviour due to GI symptoms. The aim of this study was to compare the co-morbidity and its relation to health care seeking behaviour among non-patients with persistent FGID with those who are persistently GI symptom free, in a randomly-selected adult Swedish population.

## Methods

### Setting

**Figure 1:**
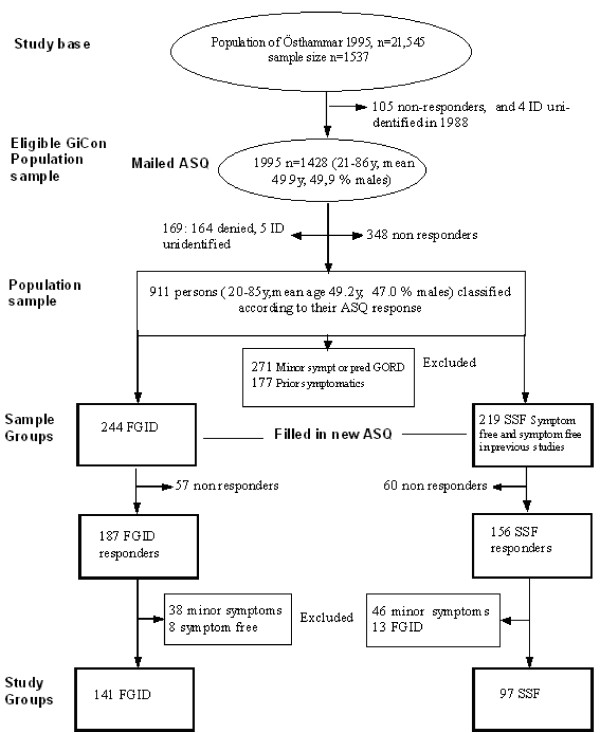
**Sampling procedure of the Gastrointestinal Consult Study (GiCon study)**. Sampling procedure of the Gastrointestinal Consult Study (GiCon study) with relation to the prior study of 1995.

In January 1995 there were a total of 21,545 Swedish citizens in the Östhammar community, 14,932 of whom were born between 1909 and 1974. Data from three prior studies[2], which included random samples of the Östhammar population, were used to provide symptom-free controls in the current study. We called this new study the Gastrointestinal Consult Study (GiCon). A sample was drawn from the National Swedish Population Registry 1995, now involving all Swedish citizens born between 1909 to 1974 and thus 20 to 87 years of age, born on day 3, 12 or 24 of each month (n = 1537), i.e. using the same date of birth criteria as in previous studies on the same population in 1988 and 1989. Thus, we increased the upper age limit to 87 years in order to include all prior participants. As a consequence of the sampling strategy, all subjects younger than 27 years of age were included for the first time (n = 86). Most the participants were now being approached for the third time. Of the 1537 subjects in the study base (see Figure 1), 105 were excluded due to having declined further participation in the 1988 study and 4 were excluded due to unidentified ID. Thus, 1428 subjects were still eligible. They were sent a validated postal questionnaire, called "The Abdominal Symptom Questionnaire" (ASQ) [21], described below. Two reminders were sent out when necessary. Drop-out reasons are shown in Figure 1, together with the mean age and sex distributions of the 911 responders who constituted the population sample of the current GiCon study.

#### Formation of the study groups and symptom classification

All 911 responders in the population sample were classified according to their response in the ASQ (see Figure 1) as having either Functional Gastrointestinal Disorder (FGID, n = 244), i.e. functional dyspepsia or irritable bowel syndrome, or being Strictly Symptom Free (SSF, n = 219). Moreover, subjects reporting minor symptoms that did not fulfil the criteria for dyspepsia or irritable bowel syndrome, or reporting isolated symptoms of gastroesophageal reflux symptoms (GERS, n = 271), were excluded. Subjects who were free from abdominal symptoms in this 1995 survey but had reported symptoms in the previous studies (1988–1989) were labelled as Prior Symptomatic (n = 177) and were also excluded.

The FGID and SSF sample groups (n = 244 + 219 = 463) were then invited by post to participate in the current investigation and to visit one of the six local health centres. Subjects who did not respond to the first letter were sent a reminder, and non-responders to the second letter were finally contacted by phone. In total, 187 (77%) with FGID and 156 (71%) with SSF accepted the invitation. There were 117 (57 FGID, 60 SSF) subjects with incomplete (n = 12) or no (n = 105) response. At the health centres, the participants filled in an ASQ and a Complaint Score Questionnaire (CSQ), as described below, and the height and weight of each was measured. The time period between the original 1995 ASQ and the second ASQ survey in 1996 ranged from 7 to 15 months. Of the FGID group of 187 subjects, 8 subjects who now reported no symptoms and 38 with minor symptoms were excluded; and out of the 156 SSF, 13 who now reported FGID and 46 with minor symptoms were also excluded. Thus, data from 141 FGID subjects (mean age 45.7 y, 34% men) and 97 SSF subjects (mean age 52.4 y, 48% men) remained in the analysis. The sampling procedure for retrospective data – twice over one year for those with, and up to four times over eight years for those without, GI symptoms – assured persistent symptomatic status.

### Questionnaires

#### The abdominal symptom questionnaire

**Figure 2:**
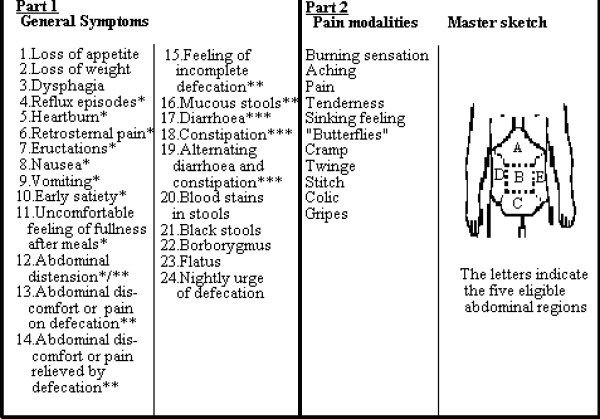
**GI symptoms, pain modalities and location**. The symptoms inquired are shown in Part 1, and the pain and discomfort modalities with the master sketch for indicating their abdominal location in Part 2. The asterisks are explained in the text. Swedish laymen terms were used in the questionnaire. The master sketch shows the eligible pain locations.

The Abdominal Symptom Questionnaire (ASQ) has been validated previously and found to be reliable and reproducible [2,13,22]. In the questionnaire, subjects were asked if they had been troubled (Yes/No) by any of the 29 listed general gastrointestinal symptoms over the previous three months. They were also asked if they had been troubled by any of 11 listed descriptors of abdominal pain and where the symptom was located (upper, centre or lower abdominal, right and left flank, as shown in Figure 2). A similar ASQ was used for the postal survey and the surgery visit, with the latter extended to include a symptom severity Likert scale graded from zero to seven for each symptom asked for. In the analysis, the data were pooled into a three-grade scale (1, 1–4, 5–7)

##### Definitions of symptoms from the ASQ

(See Figure 2) [23].

*Dyspepsia *was defined as one or more of the symptoms (reflux episodes, heartburn, retrosternal pain, nausea, vomiting, early satiety, uncomfortable feeling of fullness after meals, abdominal distension), and one or more of the pain modalities (burning sensation, aching, pain, tenderness, sinking feeling, "butterflies", cramp, twinge, stitch, colic, gripes) with any abdominal location except the lower part, but no concomitant IBS.

*Irritable Bowel Syndrome (IBS) *was defined as one or more of the symptoms (abdominal distension, abdominal discomfort or pain on defecation, abdominal discomfort or pain relieved by defecation, feeling of incomplete defecation, mucous stools) and one or more of the symptoms (diarrhea, constipation, alternating diarrhea and constipation) together with one or more of the pain modalities (burning sensation, aching, pain, tenderness, sinking feeling, "butterflies", cramp, twinge, stitch, colic, gripes), with any location.

When using these definitions of dyspepsia and IBS, responders with only reflux symptoms, i.e. heartburn and/or retrosternal pain but no abdominal pain or discomfort, were classified as having GERS and not dyspepsia, while those with such symptoms *and *abdominal pain or discomfort fell into the dyspepsia group. Also, IBS, as defined below, was given priority over dyspepsia. Thus, concomitant occurrence of both led to a diagnosis of IBS. This definition is in accordance with published guidelines [24].

##### Functional Gastrointestinal Disorder, FGID

FGID was defined as either dyspepsia or IBS.

*Minor symptoms *refer to symptoms not fulfilling the criteria of dyspepsia or IBS. By definition, those with GERS were included in this group

*"Strictly Symptom Free" *(SSF) is defined as having no reported symptoms at all in the Abdominal Symptom Questionnaire in the 1995 survey, and subjects stating that they had not even been troubled previously with abdominal symptoms. Those subjects who had participated in the two former surveys in 1988 and 1989 (n = 265) should also have reported no symptoms in each of those two investigations.

*"Prior symptomatics" *were those with no symptoms in the 1995 survey but with GI symptoms reported in the prior studies of 1988 and 1989.

#### The complaint score questionnaire

**Figure 3:**
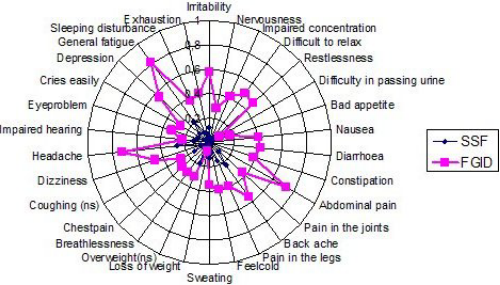
**Proportion of subjects with FGID and with SSF reporting complaints during the previous three months in the CSQ**. Proportions (0–1.0) of subjects with FGID and with SSF (n = 238) reporting complaints during the previous three months in the Complaint Score Questionnaire (CSQ). There were significance differences *(p < 0.0016) on an age and sex adjusted logistic regression for all variables except coughing (ns) and excessive weight (ns).

The Complaint Score Questionnaire contains 30 questions, as shown in Figure 3, indicating the presence or absence of 30 different symptoms [25]. The questions are dichotomous and can be categorized into six domains: abdominal/urinary, ache/fatigue, muscular-skeletal, nutritional, cardio-vascular and depressive.

#### Other questions

The participants were asked to state their coffee, alcohol and tobacco consumption, and whether they had ever had peptic ulcer disease or had ever consulted a doctor for GI complaints. Also, the past three months of general pain and all GI medication were indicated. Educational background was registered at five levels (elementary, comprehensive, secondary, upper secondary, university) and medical knowledge was evaluated by means of a self-explanatory questionnaire (see Additional File). The answers were scored with a sum of 0–15.

### Statistical power and analysis

One hundred and twenty three subjects were required (in each the FGID and SSF groups) in order to have a 90% power at the P < 0.05 level to detect a 20% absolute difference in exposure variables. This assumed a 24% prevalence of FGID in the population, equal numbers of subjects in the FGID and SSF groups, 15 and 20% absolute difference in the exposure variables within the two steps of the study, 75% response rate on the ASQ, and 25% exclusion from each group in the last step. The variables analysed are presented in the Additional File. Univariate analyses were performed using Student's t-test, Wilcoxon's ranksum test (Mann Whitney) and Pearson's chi-2 test. Multivariate analysis with FGID as the dependent variable was performed using a logistic regression, and with dyspeptic severity as the dependent variable with the ordinal logistic regression technique. Age and BMI were linear to outcome and were thus handled as continuous variables. To test the symptoms in the CSQ, we made an age and sex adjusted logistic regression model for each symptom and accepted a P value less than 0.05/(number of symptoms) = 0.05/30 ≈ 0.0016 as significant. The explanatory variables were evaluated by a logistic regression in a sex and age adjusted model.

The association between each potential determinant obtained from the questionnaires and the presence of FGID was quantified by using odds ratios and 95% confidence intervals. All exposure variables with P < 0.25 were then entered together in a multivariate logistic regression model to evaluate which was independently associated with the presence of FGID. A factor analysis was performed using all 30 complaints and factors with eigenvalues greater than 1.3. Four factors were obtained by a principal components analysis with varimax rotation. The 30 variables were dichotomized with the highest 1/3 given the value 1 and the rest the value 0. A logistic regression was performed with the four factors age and sex adjusted, and factors with p > 0.05 were excluded. To test whether the model fitted the data, a Pearson goodness-of-fit test with p values greater than 0.05 was performed. When the number of covariates approximated the number of observations, the Hosmer-Lemeshow test was performed to determine whether the model fitted the data. For the ordinal logistic model, comparison with a multinomial model made an approximate fit test. No interactions were found between the variables in the main model. Ninety five per cent confidence intervals (CI) were computed using parametric methods. A p-value of 0.05 or less was generally regarded as statistically significant. All tests were two-tailed and the statistical package Stata 8 was used for analysis [27].

### Ethics

This study was approved by the Ethics Committee of the Medical Faculty, Uppsala University, June 5th 1996.

## Results

### Representativeness of study sample

In order to investigate the effect of the drop-outs during the sampling procedure, the mean ages, sex ratios and education levels in the corresponding FGID and SSF groups were analysed as shown in Table 1, for the samples from 1988, 1995 and 1996 (see Table 1) [13]. There were no significant differences in any of these aspects (see Table 1).

**Table 1:** Comparison between previous population studies in Östhammar, Sweden. Age, sex and education level at different stages of the sampling process. From the first population sample in 1988 to the present study 1996. ns = p > 0.05.

Group (G)	Sample	Year	n	Age years mean (SD)	Sex % males	Education median level (range)
1	First population sample	1988	1156	48.9 (16.0)	50.4	3 (1–4)*
2	Eligible sample	1995	1428	49.9 (17.2)	49.9	3 (1–4)**
3	Population sample	1995	911	49.2 (16.46)	47.0	3 (1–4)
4	Sample group FGID	1995	244	45.5 (15.3)	36.1	3 (2–4)
5	Sample Group SSF	1995	219	51.7 (17.6)	51.6	3 (1–4)
6	Study Group FGID	1996	141	45.7 (14.3)	34.0	3 (2–4)
7	Study Group SSF	1996	97	52.4 (15.3)	48.0	3 (1–4)
G2 vs G3				ns	ns	
G2 vs G3				ns	ns	ns
G4 vs G6				ns	ns	ns
G5 vs G7				ns	ns	ns

*responders = 1156 **responders n = 1384

### Study group characteristics

*As shown in *Table 2, *there were more females among those with FGID. However, there were no intergroup differences in education, medical knowledge, BMI, intake of coffee, alcohol and smoking. The age difference was irrelevant, as those with SSF had been largely excluded due to prior study results. Disease-related variables were significantly different between the study groups. These variables were introduced to further modelling, as shown below. The variables 'intake of GI medicine' and 'previous PUD' were not included due to sparse data.*

**Table 2:** Comparison between explanatory variables for subjects with FGID and SSF. Comparison between explanatory variables for subjects with FGID and SSF adjusted for sex and age. Ordinal variables are presented as median (range), dichotomous variables as proportion %, and continuous variables as mean (SD).

Variable	FGID n = 141	SSF n = 97	P
AGE	45.7 (14)	52.4 (15)	**not relevant**
SEX (female %)	66	53	0.026 ***
BMI	26.3 (4.7)	26.2 (4.2)	Ns *
Education	3 (2–4)	3 (1–4)	Ns **
Medical knowledge	10 (4–15)	11 (5–15)	Ns **
GI sympt severity	4 (3–5)	0 (0–0)	<0.0005 **
GI Consultation (ever)	72%	8%	<0.0005 ***
Pain medicine (3 month)	77%	34%	<0.0005 ***
GI medicine (3 month)	32%	1%	<0.0005 ***
Previous PUD (ever)	12%	1%	0.006 ***
Coffee	2 (2–3)	2 (2–3)	Ns **
Alcohol	4 (3–4)	3 (2–4)	Ns **
Smoking	1 (1–2)	1 (1–2)	Ns **

* = Student's t-test ** = Mann-Whitney test *** = Pearson Chi-2 test

### CSQ and FGID vs. SSF

The results from the 30 CSQ complaints for the FGID and SSF study groups are presented in Figure 3. Those with persistent FGID scored statistically higher on all variables except difficulties in passing urine, excessive weight, coughing and impaired hearing.

### Risk modelling

Risks of reported CSQ complaints for FGID vs. SSF, expressed as age and sex adjusted OR, are presented in Table 3. GI complaints (abdominal pain, nausea, diarrhea and constipation) were excluded as we aimed to analyze the co-morbidity with GI symptoms. The OR was significant for all except four complaints. After adjusting for alcohol and pain and GI drug intake (Table 3), 20 complaints remained significant. A factor analysis was performed including the 26 non-GI complaints. After a varimax rotation of the four factors with eigenvalues > 1.3, we found four factors representing psychological illness, somatic illness, ache/fatigue and one "miscellaneous" (Table 3). Each factor was then introduced in a logistic regression model adjusted for sex and age (Table 3), and the "miscellaneous" factor was shown to be non-significant. In the last sequential analysis, the three factors that remained significant in the prior analysis were introduced together into a main effect model, adjusted for age and sex. The OR for these factors remained significant, as shown in Table 3.

**Table 3:** Odds ratios of FGID/SSF for complaints in the Complaints Score Questionnaire (CSQ). Odds ratios (OR, with 95% confidence intervals (CI)) of FGID/SSF (n = 238) for complaints elicited by the CSQ. Logistic regression is presented in different models. A factor analysis extracted four factors: A = psychological illness factor, B = somatic illness factor, C = miscellaneous factor, D = ache/fatigue factor. These were used in the modelling in the right two columns.

	I	II	III	IV	V
Symptom	OR (CI) by Models adjusted for sex and age	OR (CI) by Models adjusted for sex, age, alcohol, pain tablets, GI-tablets	FACTOR	OR (CI) by Models adjusted for sex and age	OR (CI) by a main effect model adjusted for sex and age

**SSF **(all variables)	1 (Reference)	1 (Reference)			
**FGID**					
Cries easily	6.7 (2.3–19.9)	9.8 (2.0–47)	A	8.0 (4.1–15.8) ^1) Psychological illness^	8.4 (4.0–17.5) ^1) Psychological illness^
Sleeping disturbance	6.2 (2.7–14.0)	3.2 (1.3–8)	A		
General fatigue	14.5 (7.4–28.7)	12.6 (5.3–30)	A, D		
Irritability	8.8 (4.1–17.8)	5.6 (2.3 – 13.7)	A, D		
Nervousness	18.4 (4.2–80.3)	14.3 (2.8 – 72)	A		
Impaired concentration	19.0 (5.7–63.8)	15.3 (4.0 – 58)	A		
Difficulty to relax	15.7 (6.0–41.5)	10.9 (3.7–32)	A		
Restlessness	40.0 (9.4–170)	32.2 (6.7–154)	A		
Depression	8.6 (4.1–18)	4.7 (2.0 – 11)	A		
Exhaustion	12.7 (4.4–37)	9.1 (2.7–30)	A		
Chest pain	40.0 (5.3–300)		B	3.7 (2.0–7.1) ^1) Somaticillness^	2.8 (1.3–5.7) ^1) Somaticillness^
Pain in the joints	6.2 (2.8–13.6)	7.5 (2.6–21)	B		
Pain in the legs	4.4 (2.2–8.9)	3.8 (1.6–9.3)	B		
Overweight	2.0 (1.0–4.0)	1.4 (0.6–3.6)	B		
Breathlessness	8.9 (3.2–25)	12.1 (3.3–45)	B		
Dizziness	10.1 (4.2–24)	11.4 (3.8–34)	B		
Impaired hearing	3.0 (1.3–6.8)	3.1 (1.0–9.5)	B		
Eye problem	4.2 (1.9–9.1)	3.4 (1.3–9.0)	B		
Loss of weight	-	-	C	1.7 (0.9–3.0) ^1) Miscellaneous^	
Bad appetite	-	-	C		
Feeling cold	7.3 (3.1–17)	7.0 (2.6–19)	C		
Difficulty in passing urine	9.6 (2.0–47)	9.1 (1.4–59)	C		
Back ache	4.4 (2.4–8.2)	2.0 (0.9–4.3)	D	2.9 (1.6–5.2) ^1) Ache/fatigue^	4.3 (2.1–8.7) ^1) Ache/fatigue^
Headache	6.3 (3.4–12)	4.1 (1.9 – 9.1)	D		
Sweating	3.6 (1.7 – 7.4)	3.3 (1.3 – 8.5)			
Coughing	2.0 (0.98–4.2)	1.7 (0.6–4.4)			

^1) ^Reference group (OR = 1) is those coded 0 in each factor

### Consulters versus non consulters

**Figure 4:**
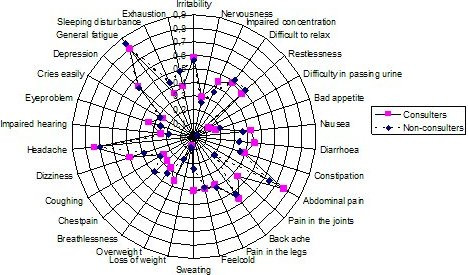
**Proportion of complaints from the Complaint Score Questionnaire among those with FGID, divided into Consulters and Non-Consulters**. Proportion (0–1.0) of complaints from the Complaint Score Questionnaire (CSQ) among those with FGID, divided into Consulters and Non-Consulters (n = 141). None of the variables showed a significant difference between Consulters and Non-Consulters for P values less than 0.0016, tested by a sex and age adjusted logistic regression.

Consulters and non-consulters among those with persistent FGID were compared regarding their complaints, as reported in the CSQ. The proportion (0–1.0) per complaint is shown in Figure 4. There were no statistically significant differences between the consulters and non-consulters for any of the complaints (adjusted for age and sex, p > 0.0016).

### ASQ symptom severity and consulting behaviour vs CSQ factors

From the ASQ, the mean grades of GI symptom severity for affirmative symptoms were analyzed against the three final CSQ factors from Table 3 (psychological illness, somatic illness, ache/fatigue) and for age, sex and consulting behaviour, as shown in Table 4. The analysis showed an obviously higher risk of increased GI symptom severity for consulters (OR 12.3) and for psychological illness (OR 4.5), while somatic illness and ache/fatigue had a low risk, with the 95% CI close to 1.0. From the ASQ, consulting behaviour was analyzed for the CSQ factors psychological illness, abdominal illness, age and sex (see Table 5). The analysis showed a greater chance of being a consulter for abdominal illness (OR 2.0) and psychological illness (OR 2.2).

**Table 4:** Odds ratios of graded GI symptom severities in the ASQ for consulting, psychological illness, somatic illness and ache/fatigue factors. Odds ratios (OR, with 95% confidence intervals (CI)) of graded (0,1,2) GI symptom severity in the ASQ for consulting, psychological illness, somatic illness and ache/fatigue factors, age and sex, for both FGID and SSF (n = 232). Ordinal logistic regression.

Variable	OR (CI)
Psychological illness low	1
high	4.5(2.4–8.4)
Somatic illness low	1
high	2.0(1.1–3.8)
Ache/fatigue low	1
high	2.1(1.1–4.2)
Consulters no	1
yes	12.3(6.3–23.9)
Age (continuous)	0.96 (0.94–0.99)
Sex female	1
male	0.9 (0.5–1.7)

**Table 5:** Odds ratio of consulting for abdominal complaints in the ASQ, 1995, for psychological illness, abdominal illness, age and sex, for both FGID and SSF. Odds ratio (OR, with 95% confidence intervals (CI) of consulting for abdominal complaints in the ASQ for 1995 for psychological illness, abdominal illness, age and sex for both FGID and SSF (n = 232). Logistic regression. Somatic illness and ache/fatigue were excluded in the final model because they showed no significance in the prior step.

Variable	OR (CI)
Psychological illness low	1
High	2.2 (1.2–4.0)
Abdominal illness low	1
High	2.0 (1.1–3.8)
Age (continuous)	1.0 (0.99–1.0)
Sex female	1
Male	0.7 (0.40–1.3)

## Discussion

This study shows that among subjects with longstanding FGID, there is a remarkably high prevalence of psychological illness and also of non-GI somatic complaints. These are present regardless of whether the subjects have consulted their doctor about their GI problems, and are more severe in subjects with persistent FGID. Although FGID was more common in women, the consultation rates in sufferers were similar for males and females and were not age-related. Only about a quarter of the sufferers had never consulted their doctor.

We consider that our findings can be generalized to the whole population, as the study groups were sampled from a well-defined and thoroughly investigated population, most of whom had participated in prior studies [28,29]. The original study base was formed in 1988 from the Swedish National Population register, which guarantees complete coverage of all citizens. There were no differences in age, gender or education level between the study and the sample groups or between the population samples from 1988 and 1995. Also, the proportions of those reporting symptoms explicable in terms of an organic disease have been shown to be insignificant [13]. Thus, the FGID subjects and the symptom-free subjects would seem to be representative.

The validity of the research tool is a potential source of bias. However, for symptom reporting, only well-validated questionnaires were used. Both the ASQ and the CSQ have been adequately validated [28-30]. The psychological illness factor identified in this study embraces symptoms of both "neurotic" and "personality" dimensions, listed in Table 3, and the outcome seems plausible. The questionnaire used to assess medical knowledge was simple and straightforward, with kappa values per question 1 = 0.70, 2 = 0.89, 3 = 0.47, 4 = 0.78, 5 = 0.80, 6 = 0.70 when repeated within a week, considered acceptable for all with some reservation in the 0.47 case [53].

The definitions of dyspepsia and IBS used in this study were those used in the original study from 1988 [31], when the Rome II criteria [11] were unavailable. We opted to retain our original study definitions despite ongoing changes.

Our definition of dyspepsia was more restricted in terms of symptoms than the Rome II definition, but wider in terms of abdominal location, as not only epigastric but also mid abdominal symptoms were included. The IBS definition used was in accordance with the Rome II criteria [22]. Consequently, we consider the overall prevalence of FGID in this study and the concordance on an individual level to be applicable within today's definitions [11].

Subjects with FGID were on average younger than controls without FGID, which may be expected as the prevalence of dyspepsia and IBS is generally higher in younger age groups [32]. This study was not a case control study, however, but a study of all subjects with FGID and SSF within the cohort. Any differences caused by this grouping strategy were controlled for in the analysis by gender- and age-adjusted logistic regression. There was a particular association between FGID and psychological illness, although "fibromyalgia-like" symptoms (ache and fatigue) [49] and other somatic complaints were also common. Previous outpatient studies have shown that IBS is associated with psychiatric illnesses such as depression [34], dysthymia [35] and anxiety [36], and similar findings are reported for dyspepsia [50,51]. Greater mental pathology has been reported for consulters, i.e. patients, than non-consulters with IBS [44,45,52], but this has not been demonstrated convincingly in dyspepsia subjects [53].

Healthcare seeking behaviour is complex and until now it has been studied largely in patient samples. Nyrén *et al*. found that patients with non-ulcer dyspepsia had an excessive need for sick leave compared with ulcer patients, but that the predominant reason for leave was related to musculoskeletal rather than abdominal symptoms [41]. Kettell *et al*. [42] found that severity of abdominal pain and anxiety about the seriousness of their condition were important factors in patients consulting for IBS [42]. There seem to be no differences in the use of healthcare services or co-morbidity status between the year before and the year after diagnosis of IBS [43,46].

Although this study was focused on total co-morbidity, in terms of consultation, other factors associated with FGID consultation were also considered. The total consultation pattern for the subjects will be published elsewhere. In essence, effective treatment of patients with FGID involves not only addressing GI discomfort, but also considering mental and somatic complaints such as depression and exhaustion.

## Conclusion

In the present study, psychological illness proved to be an important co-morbidity factor among subjects with FGID, and the severities of the two were linked. We cannot conclude anything about the cause of the relationship. The presence of psychological illness was also associated with a greater need for medical consultation. Fear of life-threatening illness has been reported to be an important reason for consultation in FGID [1], and this worry and anxiety needs to be taken into account when attempting to manage FGID successfully. Somatic co-morbidity was found to be a less important reason for consultation, although a high proportion of subjects with FGID (77%) in our study were taking analgesics while only 32% used specific gastrointestinal medication.

## Competing interests

The author(s) declare that they have no competing interests.

## Authors' contributions

TÅ participated in the study design and checking the coding of the data, carried out the construction of the "medical knowledge questionnaire" including a test-retest survey, distributed and collected the questionnaires, called the participants to his health centre, supported the other health centers personnel, performed all the statistical analysis, participated in writing the manuscript, and submitted the final manuscript. LA carried out the study design and contributed to the statistical analysis and the writing of the manuscript. KS contributed to checking the coding of the data and participated in the study design. SJ contributed help with the statistical analysis.

## Pre-publication history

The pre-publication history for this paper can be accessed here:



## References

[B1] Jones R, Lydeard SE, Hobbs FD, Kenkre JE, Williams EI, Jones SJ, Jones, Repper JA, Caldow JL, Dunwoodie WM, Bottomley JM (1990). Dyspepsia in England and Scotland. Gut.

[B2] Agreus L, Svardsudd K, Talley NJ, Jones MP, Tibblin G (2001). Natural history of gastroesophageal reflux disease and functional abdominal disorders: a population-based study. Am J Gastroenterol.

[B3] Agreus L (2002). Natural history of dyspepsia. Gut.

[B4] Talley NJ, Zinsmeister AR, Schleck CD, Melton LJ (1992). Dyspepsia and dyspepsia subgroups: a population-based study. Gastroenterology.

[B5] Talley N, Boyce P, Jones M (1998). Identification of upper and lower gastrointestinal symptom groupings in an urban population. Gut.

[B6] Agréus L, Talley NJ (1998). Dyspepsia: current understanding and management. Annu Rev Med.

[B7] McDougall NI, Johnston BT, Kee F, Collins JS, McFarland RJ, Love AH (1996). Natural history of reflux oesophagitis: a 10 year follow up of its effect on patient symptomatology and quality of life. Gut.

[B8] Agreus L, Borgquist L (2002). The cost of gastro-oesophageal reflux disease, dyspepsia and peptic ulcer disease in Sweden. Pharmacoeconomics.

[B9] Leong SA, Barghout V, Birnbaum HG, Thibeault CE, Ben-Hamadi R, Frech F, Ofman JJ (2003). The economic consequences of irritable bowel syndrome: a US employer perspective. Arch Intern Med.

[B10] Drossman DA, Corazziari E, Thompson WG, Talley NJ, Whitehead W (2000). The Functional Gastrointestinal Disorders.

[B11] Talley N, Stanghellini V, Heading R, Koch K, Malagelada J, Tytgat G (1999). Functional Gastroduodenal Disorders. Gut.

[B12] Whitehead WE, Gibbs NA, Li Z, Drossman DA (1998). Is functional dyspepsia just a subset of the irritable bowel syndrome?. Baillieres Clin Gastroenterol.

[B13] Agreus L, Svardsudd K, Nyren O, Tibblin G (1995). Irritable bowel syndrome and dyspepsia in the general population: overlap and lack of stability over time. Gastroenterology.

[B14] Agreus L (1993). Socio-economic factors, health care consumption and rating of abdominal symptom severity. A report from the abdominal symptom study. Fam Pract.

[B15] Haycox A, Einarson T, Eggleston A (1999). The health economic impact of upper gastrointestinal symptoms in the general population: results from the Domestic/International Gastroenterology Surveillance Study (DIGEST). Scand J Gastroenterol Suppl.

[B16] Westbrook JI, McIntosh J, Talley NJ (2000). Factors associated with consulting medical or non-medical practitioners for dyspepsia: an Australian population-based study. Aliment Pharmacol Ther.

[B17] Nyren O, Lindberg G, Lindstrom E, Marke LA, Seensalu R (1992). Economic costs of functional dyspepsia. Pharmacoeconomics.

[B18] Stanghellini V (1999). Relationship between upper gastrointestinal symptoms and lifestyle, psychosocial factors and comorbidity in the general population: results from the Domestic/International Gastroenterology Surveillance Study (DIGEST). Scand J Gastroenterol Suppl.

[B19] Haug TT, Mykletun A, Dahl AA (2002). Are anxiety and depression related to gastrointestinal symptoms in the general population?. Scand J Gastroenterol.

[B20] Koloski NA, Talley NJ, Boyce PM (2002). Epidemiology and health care seeking in the functional GI disorders: a population-based study. Am J Gastroenterol.

[B21] Agreus L, Svardsudd K, Nyren O, Tibblin G (1993). Reproducibility and validity of a postal questionnaire. The abdominal symptom study. Scand J Prim Health Care.

[B22] Agreus L, Talley NJ, Svardsudd K, Tibblin G, Jones MP (2000). Identifying dyspepsia and irritable bowel syndrome: the value of pain or discomfort, and bowel habit descriptors [In Process Citation]. Scand J Gastroenterol.

[B23] Agréus L, Svärdsudd K, Tibblin G, Nyrén O (1991). The epidemiology of dyspepsia: Symptom clusters and demographic characteristics. Gastroenterology.

[B24] Talley NJ (1991). Working Team Report. Functional Dyspepsia: A Classification with Guidelines for Diagnosis and Management. Gastroenterology International.

[B25] Tibblin G, Tibblin B, Peciva S, Kullman S, Svardsudd K (1990). "The Goteborg quality of life instrument" – an assessment of well-being and symptoms among men born 1913 and 1923. Methods and validity. Scand J Prim Health Care Suppl.

[B26] World Health Organization (1997). WHO/46 Press Release: World Health Organization.

[B27] StataCorp (2003). Stata Statistical Software: Release 80.

[B28] Agréus L, Svärdsudd K, Nyrén O, Tibblin G (1993). Reproducibility and validity of a postal questionnaire. The abdominal symptom study. Scand J Prim Health Care.

[B29] Agréus L (1993). The Abdominal Symptom Study. An Epidemiological Survey of Gastrointestinal and Other Abdominal Symptoms in the Adult Population of Östhammar, Sweden. PhD thesis.

[B30] Tibblin G, Cato K, Svärdsudd K (1990). Goteborg quality of life study of men born in 1913 and 1923 – age, sex, job satisfaction and cardiovascular diseases. Scand J Prim Health Care.

[B31] Agréus L, Nyrén O, Svärdsudd K, Tibblin G (1990). Ont i magen-En epidemiologisk studie om bukbesvär i Östhammars kommun. Svenska Läkarsällskapets Hygea.

[B32] Agréus L, Svärdsudd K, Talley NJ, Jones MP, Tibblin G (2001). Natural history of gastroesophageal reflux disease and functional abdominal disorders: a population-based study. Am J Gastroenterol.

[B33] Gupta S, Masand PS, Kaplan D, Bhandary A, Hendricks S (1997). The relationship between schizophrenia and irritable bowel syndrome (IBS). Schizophr Res.

[B34] Masand PS, Kaplan DS, Gupta S, Bhandary AN, Nasra GS, Kline MD, Margo KL (1995). Major depression and irritable bowel syndrome: is there a relationship?. J Clin Psychiatry.

[B35] Masand PS, Kaplan DS, Gupta S, Bhandary AN (1997). Irritable bowel syndrome and dysthymia. Is there a relationship?. Psychosomatics.

[B36] Lydiard RB, Fossey MD, Marsh W, Ballenger JC (1993). Prevalence of psychiatric disorders in patients with irritable bowel syndrome. Psychosomatics.

[B37] Sullivan G, Blewett AE, Jenkins PL, Allison MC (1997). Eating attitudes and the irritable bowel syndrome. Gen Hosp Psychiatry.

[B38] Walker EA, Gelfand AN, Gelfand MD, Green C, Katon WJ (1996). Chronic pelvic pain and gynecological symptoms in women with irritable bowel syndrome. J Psychosom Obstet Gynaecol.

[B39] Woodman CL, Breen K, Noyes R, Moss C, Fagerholm R, Yagla SJ, Summers R (1998). The relationship between irritable bowel syndrome and psychiatric illness. A family study. Psychosomatics.

[B40] Sperber AD, Atzmon Y, Neumann L, Weisberg I, Shalit Y, M Abu-Shakrah, Fich A, Buskila D (1999). Fibromyalgia in the irritable bowel syndrome: studies of prevalence and clinical implications. Am J Gastroenterol.

[B41] Nyrén O, Adami HO, Gustavsson S, Lööf L (1986). Excess sick-listing in nonulcer dyspepsia. J Clin Gastroenterol.

[B42] Kettell J, Jones R, Lydeard S (1992). Reasons for consultation in irritable bowel syndrome: symptoms and patient characteristics. Br J Gen Pract.

[B43] Ruigomez A, Wallander MA, Johansson S, Garcia Rodriguez LA (1999). One-year follow-up of newly diagnosed irritable bowel syndrome patients. Aliment Pharmacol Ther.

[B44] Ballenger JC, Davidson JR, Lecrubier Y, Nutt DJ, Lydiard RB, Mayer EA (2001). Consensus statement on depression, anxiety, and functional gastrointestinal disorders Irritable bowel syndrome, anxiety, and depression: what are the links?. J Clin Psychiatry.

[B45] Lydiard RB (2001). Irritable bowel syndrome, anxiety, and depression: what are the links?. J Clin Psychiatry.

[B46] Whitehead WE, Palsson O, Jones KR (2002). Systematic review of the comorbidity of irritable bowel syndrome with other disorders: what are the causes and implications?. Gastroenterology.

[B47] Jarrett ME, Burr RL, Cain KC, Hertig V, Weisman P, Heitkemper MM (2003). Anxiety and depression are related to autonomic nervous system function in women with irritable bowel syndrome. Dig Dis Sci.

[B48] Aro P, Ronkainen J, Storskrubb T, Bolling-Sternevald E, Talley NJ, Agréus L (2001). Prevalence of symptoms and upper endoscopic findings in a random adult population. A report from Kalixanda study. Gastroenterology.

[B49] Wolfe F, Smythe HA, Yunus MB, Bennett RM, Bombardier C, Goldenberg DL, Tugwell P, Campbell SM, Abeles M, Clark P (1990). The American College of Rheumatology 1990 Criteria for the Classification of Fibromyalgia. Report of the Multicenter Criteria Committee. Arthritis Rheum.

[B50] Talley NJ, Fung LH, Gilligan IJ, McNeil D, Piper DW (1986). Association of anxiety, neuroticism, and depression with dyspepsia of unknown cause. A case-control study. Gastroenterology.

[B51] Kane F, Strohlein J, Harper RG (1993). Nonulcer dyspepsia associated with psychiatric disorder. South Med J.

[B52] Drossman DA, McKee DC, Sandler RS, Mitchell CM, Cramer EM, Lowman BC, Burger AL (1988). Psychosocial factors in the irritable bowel syndrome. A multivariate study of patients and nonpatients with irritable bowel syndrome. Gastroenterology.

[B53] Sackett DL (1992). A primer on the precision and accuracy of the clinical examination. JAMA.

